# The role of preoperative iron deficiency in colorectal cancer patients: prevalence and treatment

**DOI:** 10.1007/s00384-017-2898-1

**Published:** 2017-09-09

**Authors:** M. J. Wilson, J. W. T. Dekker, J. J. Harlaar, J. Jeekel, M. Schipperus, J. J. Zwaginga

**Affiliations:** 1 TRIP Hemovigilance and Biovigilance Office, Leiden, the Netherlands; 2 Department of Surgery, Erasmus University Medical Center Rotterdam, Rotterdam, the Netherlands; 3 Department of Surgery, Reinier de Graaf Hospital, Delft, the Netherlands; 4 Department of Surgery, Westfriesgasthuis Hoorn, Hoorn, the Netherlands; 5 Department of Surgery, VU Medical Center Amsterdam, Amsterdam, the Netherlands; 6 Department of Neuroscience, Erasmus University Medical Center Rotterdam, Rotterdam, the Netherlands; 7 Department of Hematology, Haga Teaching Hospital, The Hague, the Netherlands; 8 Sanquin Research, Center for Clinical Transfusion Research, Leiden, the Netherlands; 9 Department of Immunohematology and Blood Transfusion, Leiden University Medical Center, Leiden, the Netherlands

**Keywords:** Preoperative anaemia, Iron status, Colorectal cancer, Patient blood management

## Abstract

**Background:**

In preoperative blood management of colorectal cancer patients, intravenous iron therapy is increasingly used to treat anaemia and prevent red blood cell transfusions. However, while iron deficiency is the most common cause of anaemia, little is known about the prevalence and namely type of iron deficiency in this population, whereas both types of iron deficiency (i.e. absolute and functional iron deficiency) are recommended to be treated differently by international cancer guidelines.

**Objective:**

The aim of present study is to investigate the prevalence and namely type of iron deficiency in colorectal cancer patients, and to assess its clinical relevance.

**Methods:**

Preoperative iron status, clinical parameters (i.e. age, ASA classification, tumour location, tumour stage) and postoperative complications were retrospectively collected for all newly diagnosed colorectal cancer patients in our institution over a 3-year period.

**Results:**

Iron deficiency was observed in 163 (48.1%) of 339 patients. Of these iron-deficient patients, 3.7% had an isolated absolute iron deficiency (AID) and 15.3% a functional iron deficiency (FID), while the rest had a combination of AID and FID. Anaemia was present in 66.1% of iron-deficient patients. Iron deficiency was significantly associated with an increased postoperative complication rate (univariable OR 1.94, *p* = 0.03, multivariable OR 1.84, *p* = 0.07), with right-sided tumours (*p* < 0.001), high ASA classification (*p* = 0.002), advanced tumour stage (*p* = 0.01) and advanced age (*p* = 0.04). In comparing clinical parameters between patients with AID and FID, advanced age was significantly associated with FID (*p* = 0.03), and the presence of anaemia with AID (*p* = 0.02).

**Conclusion:**

In preoperative colorectal cancer patients, there is a high prevalence of iron deficiency, including a high percentage of patients with—a component of—functional iron deficiency, associated with the increased postoperative complication rate. As both types of iron deficiency require a different treatment strategy, our results illustrate the therapeutic potential of especially intravenous iron supplementation in patients with severe iron deficiency and stress the urgency of routinely monitoring preoperative iron status and differentiation between types of iron deficiency. As iron therapy may also be potentially harmful in respect to stimulation of tumour growth, future clinical trials assessing the long-term effect of iron therapy are necessary.

## Introduction

Preoperative anaemia is frequently observed in colorectal cancer patients, with reported case incidences of > 30% [1]. Preoperative anaemia generally is associated with increased postoperative morbidity and mortality [2] and is also reported to be a cause of inferior long-term outcome, possibly by worsening of tumour hypoxia [3, 4]. Furthermore, preoperative anaemia is associated with increased utilisation of allogeneic red blood cell (RBC) transfusion, which, for its part, is also associated with deleterious effects on the short- and long-term outcome in colorectal cancer patients [5, 6].

Iron deficiency (ID) is the most common cause of preoperative anaemia in colorectal cancer patients [7]. Contributing mechanisms to the development iron deficiency anaemia include chronic tumour-induced blood loss and impaired iron homeostasis associated with chronic disease. While chronic blood loss will deplete iron stores and cause absolute iron deficiency (AID), functional iron deficiency (FID) is characterised by both reduced iron uptake in the gut and sequestration in the reticulo-endothelial system of absorbed iron, resulting in a reduction of biologically available iron [8]. Next to AID, FID is the second most prevalent cause of anaemia. FID is especially known from patients with immune activation and therefore termed as anaemia of inflammation of anaemia of chronic disease.

The importance of this differentiation lies in the fact that the indication for initiation and the administration route of iron therapy differ between AID and FID [9]. In patients with AID, iron therapy is recommended to be started independently of the actual haemoglobin (Hb) level, while in patients with FID, iron therapy is advised only if patients are symptomatic because of iron deficiency and/or anaemia and should be withheld in patients with high ferritin levels (i.e. > 1000 ng/mL). In addition, in patients with FID, oral iron is poorly absorbed in the duodenum, while intravenous iron is more effective. On the other hand, restrictive iron therapy might be advisable for cancer patients in general, as iron is reported to stimulate tumour growth. The latter could be even more important for cancer patients with FID. This cancer-induced immune response namely might well protect against proliferation of tumour cells [8, 10].

Notwithstanding possible detrimental effects, iron in preoperative blood management to reverse the anaemia-associated prognosis has gained more attention [11]. In particular, this has led to the increased use of preoperative intravenous iron supplementation. Whereas preoperative anaemia is a well-known and frequent complication in colorectal cancer patients, little is known about the prevalence of iron deficiency [7, 12]. While research is being carried out on the efficacy of preoperative oral and intravenous iron therapy in patients with iron deficiency anaemia, no trials differentiate between AID and FID and often only the Hb increase and reduction in RBC transfusions are studied [13, 14].

Despite the recommendations by international oncological guidelines [15, 16], routinely monitoring preoperative iron status is often not standard of care and is, for example, not incorporated into the Dutch guideline on the treatment of anaemia in oncological patients. The aim of present study is to identify the prevalence and type of iron deficiency, and to assess the clinical relevance of iron deficiency.

## Methods

All patients undergoing resection for colorectal cancer between 1 July 2013 and 1 July 2016 at the Department of Surgery, Reinier de Graaf Hospital, were eligible for inclusion. In these patients, the inclusion criterion was the availability of iron status (i.e. iron, transferrin, transferrin saturation, ferritin), measured directly after colonoscopy and suspicion of colorectal cancer. Clinical and pathological data, including age, gender, ASA classification, tumour type, pathological tumour stage, neoadjuvant treatment and 30-day overall postoperative complications (i.e. pulmonic, cardiologic, thrombotic, infectious, neurologic), were collected by the Dutch Surgical Colorectal Audit (DSCA), a disease-specific national audit. This audit collects information on patient, tumour, treatment, and 30-day and in-hospital outcome characteristics of all patients undergoing a resection for primary colorectal carcinoma in the Netherlands. The data set is based on evidence-based guidelines and is cross-checked on a yearly basis with data from the Netherlands Cancer Registry. In addition, haemoglobin values (i.e. at diagnosis, preoperative and postoperative), and iron status at diagnosis, were retrospectively collected.

According to the World Health Organization (WHO), anaemia was defined as Hb < 8 mmol/L in men and < 7.5 mmol/L in women. Iron deficiency was defined as transferrin saturation (TSAT) < 20% and was further classified as AID, FID or a combination of both conditions. AID was defined as TSAT < 20% and increased transferrin (> 3.6 g/L) and FID as TSAT < 20%, reduced to normal transferrin and increased ferritin (> 200 μg/L).

Tumour locations were classified as the right colon (i.e. cecum, colon ascendens, hepatic flexure), transverse colon, left colon (i.e. splenic flexure, colon descendens, sigmoid) and rectum. Tumour staging and tumour grading were determined according to the AJCC recommendations in colorectal cancer and were given by pathologic examination. The ASA physical status classification system was used for assessing the fitness of patients before surgery.

The results are mainly illustrated by descriptive statistics. *χ* ^2^, Fisher’s exact and Student’s *t* tests were used to compare the frequencies of both categorical and continuous variables with iron status (i.e. iron deficiency versus non-iron deficiency and absolute versus functional iron deficiency) and tumour location (i.e. colon versus rectum). Binary logistic regression analysis was performed to identify the relationship between iron deficiency at diagnosis and postoperative complication. All variables in the univariable analysis were included in the multivariable analysis. A significance level of 0.05 was considered to be statistically significant.

Approval by the local medical ethics committee was obtained. Our institution, a teaching hospital, is making use of opt-out consent. Each included patients had given consent by not declining to give consent.

## Results

### Incidence of iron deficiencies

**Fig. 1:**
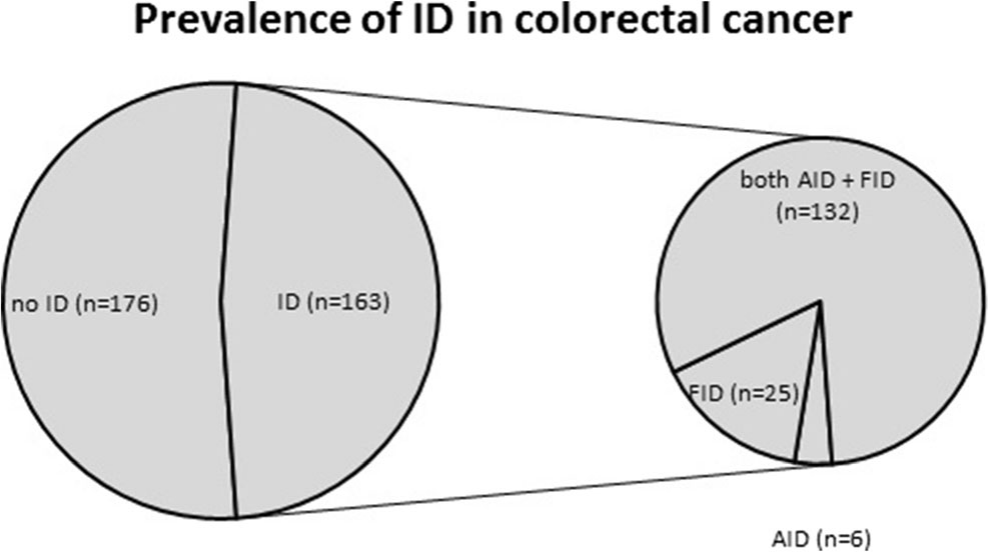
Prevalence of iron deficiency

In total, 429 patients underwent surgery for colorectal cancer, and iron status was available in 339 patients (all measured at diagnosis). Table 1 shows the baseline characteristics of included patients. The mean age at presentation was 69.6 (range 28–95); 185 males and 154 females were included. Most patients (58.1%) were classified as ASA 2 and the most frequent site of tumour occurrence was the left colon (36.6%), followed by the rectum (29.5%), right colon (25.4%) and transverse colon (8.6%). The majority of patients were classified as pTNM stage 2 (33.6%), followed by stage 1 (29.8%), stage 3 (28.0%) and stage 4 (8.6%). Of 339 patients, preoperatively, 35 patients (10.3%) received radiotherapy alone, 19 patients (5.6%) received concomitant chemoradiotherapy and 8 patients (2.4%) received chemotherapy alone. In total, 256 patients (79.0%) were symptomatic at presentation; most patients presented with blood loss (*n* = 108), followed by change in stool (*n* = 72), other (*n* = 43) (i.e. abdominal pain, weight loss, fatigue) and anaemia (*n* = 33). Iron deficiency was observed in 163 patients (48.1%) and anaemia in 115 patients (33.9%). Among these iron-deficient patients, 6 (3.7%) and 25 (15.3%) patients were absolute and functional iron deficient, respectively. In the majority of patients (*n* = 132; 81.0%), iron deficiency was caused by a combination of AID and FID. In total, 80% of anemic patients had some form of iron deficiency (5.2% AID, 9.6% FID, 65.2% combination AID and FID). Of non-anemic patients, 14 (6.3%) were functional iron deficient, and 57 (25.4%) had a combination of AID and FID; no patients were absolute iron deficient (Fig. 1).

**Table 1:** Patient baseline characteristics (*n* = 339)

	Number	Percent
Gender		
Male	185	54.6
Female	154	45.4
Age (years)		
Mean (range)	69.63 (28–95)	
ASA classification		
I	76	22.4
II	197	58.1
III	65	19.2
IV	1	0.3
Tumour location		
Right colon	86	25.4
Transverse colon	29	8.6
Left colon	124	36.6
Rectum	100	29.5
Neoadjuvant treatment		
Chemotherapy	8	2.4
Radiotherapy	35	10.3
Concomitant chemoradiotherapy	19	5.6
None	277	81.7
pTNM stage^a^		
I	101	29.8
II	114	33.6
III	95	28.0
IV	29	8.6
Presenting symptoms		
Asymptomatic (population screening)	68	21.0
Symptomatic	256	
Blood loss	108	33.3
Change in stool	72	22.2
Workup of anaemia	33	10.2
Other	43	13.3
Unknown	15	
Iron deficiency		
No	176	51.9
Yes	163	48.1
Absolute iron deficiency	6	3.7
Functional iron deficiency	25	15.3
Both conditions	132	81.0
Anaemia at presentation		
No	224	66.1
Yes	115	33.9
Absolute iron deficiency	6	5.2
Functional iron deficiency	11	9.6
Both conditions	75	65.2

^a^After chemo and/or radiotherapy in 62 patients

### Associations between iron deficiency and patient and tumour characteristics

In Table 2, the proportion of patients with and without iron deficiency is given in relation to gender, age, ASA classification, tumour location, pTNM stage and the presence of anaemia. Iron deficiency was significantly more likely to occur in the right colon (*p* < 0.001), in patients with a more advanced pTNM stage (*p* = 0.01), in patients with a higher ASA classification (*p* = 0.002) and in patients with more advanced age (*p* = 0.043). Moreover, anaemia was significantly more observed in iron-deficient patients (*p* < 0.001). Gender did not show a significant association with the presence of iron deficiency. Iron-deficient patients presented more often in the workup of anaemia, as compared to non-iron-deficient patients (16.2 versus 4.7%), while non-iron-deficient patients more often were diagnosed due to the screening program.

**Table 2:** Characteristics non-iron deficiency versus iron deficiency

	Non-iron deficiency	Iron deficiency	*p* value
Number, *n* (%)	176	163	
Gender, %			0.13
Male	58.5	50.3	
Female	41.5	49.7	
Age (years)			0.043
Mean ± SD	68.5 ± 10.86	70.8 ± 10.56	
ASA, %			0.002
I + II	86.9	76.3	
III + IV	13.1	26.4	
Tumour location, %			< 0.001
Right colon	13.6	38	
Transverse colon	8.5	8.6	
Left colon	39.8	33.1	
Rectum	38.1	20.2	
pTNM stage, %			0.01
I	36.9	22.1	
II	27.3	40.5	
III	28.4	27.6	
IV	7.4	9.8	
Anaemia at diagnosis, *n* (%)	23 (13.1)	92 (56.4)	< 0.001
Presenting symptoms, *n* (%)			< 0.001
Asymptomatic	47 (27.6)	21 (13.6)	
Symptomatic	123	133	
Blood loss	60 (35.3)	48 (31.2)	
Change in stool	41 (24.1)	31 (20.1)	
Workup of anaemia	8 (4.7)	25 (16.2)	
Other	14 (8.2)	29 (18.8)	

In Table 3, the mentioned variables (i.e. gender, age, tumour location, ASA classification, pTNM stage and anaemia) were compared between patients with AID and those with FID. Results showed that advanced age was significantly associated with FID (*p* = 0.03), while the presence of anaemia was significantly associated with AID (*p* = 0.02). Gender, tumour location, ASA classification and pTNM stage were not found to have any significant relationship with AID or FID.

**Table 3:** Characteristics in absolute versus functional iron deficiency

	Absolute iron deficiency	Functional iron deficiency	*p* value
Number, *n*	6	25	
Gender, %			0.79
Male	66.7	72	
Female	33.3	28	
Age (years)			0.03
Mean ± SD	68.5 ± 4.23	74.2 ± 7.40	
Tumour location, %			0.64
Colon	83.3	68.0	
Rectum	16.7	32.0	
ASA, %			0.60
I + II	66.7	80.0	
III + IV	33.3	20.0	
pTNM stage, %			0.66
I + II	66.7	52.0	
III + IV	33.3	48.0	
Anaemia, %			0.02
No	0	56.0	
Yes	100	44.0	

### Association between iron deficiency and postoperative complication

In Table 4, the association between iron deficiency and postoperative complications is assessed by uni- and multivariable logistic regression analysis. In total, postoperative complications were observed in 75 of 339 patients. Initially, in univariable analysis, the categorical variable of severity of iron deficiency was included (i.e. no iron deficiency versus mild iron deficiency (TSAT < 20%) versus severe iron deficiency (TSAT < 10%)). As merely severe iron deficiency appeared to be significantly associated with postoperative complications (OR 1.92, *p* = 0.045, versus mild iron deficiency OR 0.97, *p* = 0.92), severe iron deficiency was included in uni- and multivariable logistic regression analyses, as shown in Table 4. In univariable analysis, severe iron deficiency was significantly associated with postoperative complications (OR 1.94, *p* = 0.030). No significant result was found in multivariable analysis (OR 1.84, *p* = 0.074).

**Table 4:** Univariable and multivariable logistic regression analysis for risk factors of postoperative complications

	Univariable			Multivariable		
	OR	95% CI	*p* value	OR	95% CI	*p* value
Age (years)	1.02	0.99–1.05	0.074	1.01	0.99–1.04	0.336
Gender						
Female versus male	0.38	0.22–0.67	0.001	0.38	0.21–0.68	0.001
ASA classification						
III–IV versus I–II	2.08	1.15–3.76	0.016	1.71	0.87–3.36	0.118
Surgery						
Laparoscopic versus open	0.34	0.17–0.68	0.002	0.29	0.13–0.62	0.002
Tumour localisation						
Rectum versus colon	1.47	0.86–2.53	0.16	2.07	1.12–3.82	0.021
Severe iron deficiency at diagnosis	1.94	1.07–3.54	0.030	1.84	0.94–3.60	0.074

### Distinction between colon and rectum tumours

In Table 5, the different variables between colon and rectum tumours are shown. Anaemia, both at diagnosis, preoperative and postoperative, was more prevalent in colon tumours (*p* < 0.001, *p* < 0.001, *p* = 0.04, respectively). Reduced Hb levels at diagnosis, preoperative and postoperative were found to be significantly associated with colon tumours (all *p* < 0.001), while a reduction in the Hb level due to surgery was more pronounced in patients with rectum tumours as compared to those with colon tumours (1.09 versus 0.96 mmol/L, *p* = 0.05). Mean duration from diagnosis to surgery was 7.4 weeks for all colorectal tumours but was significantly different for colon cancer patients (5.1 weeks) as compared to patients with rectum cancer (12.2 weeks).

**Table 5:** Characteristics in colon versus rectum cancer patients

	Colon	Rectum	*p* value
Number, *n*	239	100	
Age (years)			0.15
Mean ± SD	70.2 ± 10.4	68.3 ± 11.6	
Anaemia at diagnosis, %	41.8	15.0	< 0.001
Preoperative anaemia, %	45.1	20.6	< 0.001
Postoperative anaemia, %	76.2	65.0	0.04
Hb at diagnosis^a^			
Mean ± SD	7.78 ± 1.4	8.54 ± 1.0	< 0.001
Preoperative Hb			
Mean ± SD	7.87 ± 1.2	8.45 ± 0.9	< 0.001
Postoperative Hb			
Mean ± SD	6.91 ± 1.1	7.34 ± 0.9	< 0.001
Reduction in Hb level due to surgery			
Mean ± SD	0.96 ± 0.6	1.09 ± 0.5	0.05

^a^In mmol/L

## Discussion

The present study firstly shows a high prevalence of preoperative ID in colorectal cancer patients. Almost half of the patients with newly diagnosed colorectal cancer are iron deficient at presentation. Interestingly, most patients have isolated FID (15%) or a combination of FID and AID (81%), compared to only 4% with isolated AID. From these results, we may conclude that the high percentage of patients with FID or a component of FID suggests that inflammation plays an important role in the development of iron deficiency in colorectal cancer patients. Secondly, patients with an advanced tumour, advanced age, a tumour in the right colon and a high ASA classification are more prone to develop iron deficiency. Thirdly, iron deficiency clearly plays a role in 80% of anemic patients (5.2% AID, 9.6% FID, 65.2% combined AID and FID); however, iron deficiency is also encountered in 32% of non-anemic patients (6.3% FID, 25.4% combined AID and FID).

In addition to the high prevalence of iron deficiency, the clinical relevance of iron deficiency is studied in the present study. Particularly, in univariable analysis, severe iron deficiency is significantly associated with an increased postoperative complication rate. Despite the fact that in the present cohort, loss of significance is observed in multivariable analysis, most likely due to the relative small sample size, iron deficiency still seems to be independently associated with postoperative complications. Previous published studies namely have demonstrated the efficacy of preoperative iron supplementation with regard to reduction of the need for blood transfusion and reduction of hospital length of stay [17, 18]. In addition, lower total numbers of postoperative complications were found. These results implicate iron deficiency as an attractive treatment target to at least ameliorate short-term outcomes.

Preoperative anaemia is emerging as an important health problem in colorectal cancer patients. Importantly, preoperative anaemia has already been associated with increased short-term postoperative morbidity and mortality (< 30 days) [2, 19] and worse colorectal tumour prognosis [3, 4, 20]. Whereas preoperative anaemia is often associated with iron deficiency, up to now, guidelines for the management of cancer or chemotherapy-induced anaemia make only a few remarks on the management of iron deficiency.

The *ASCO* (American Society of Clinical Oncology) guideline [21] on *the use of epoetin and darbepoietin in adult patients with cancer* recommends to only start iron supplementation in order to improve the efficacy of erythropoietin-stimulating agents (ESAs), and to monitor iron status during the course of ESA therapy. The *ESMO* (European Society for Medical Oncology) guideline [16] states that intravenous iron therapy is more effective in terms of Hb optimisation as compared to oral iron therapy and that iron therapy seems to reduce the total number of patients receiving blood transfusions. Most elaborate is the *NCCN* (National Comprehensive Cancer Network) guideline [15] on *cancer- and chemotherapy-induced anaemia* that recommends to start iron monotherapy in absolute iron deficiency patients, independently of the presence of anaemia, to start iron therapy in patients receiving ESA, and to withheld iron therapy in patients with active infections. The NCCN guideline additionally briefly addresses treatment of merely iron deficiency in non-anemic patients. This seems to be clinically relevant as iron deficiency itself, in the absence of anaemia, can cause symptoms as impaired physical function and fatigue [22, 23]. The observed high prevalence of iron deficiency in colorectal cancer patients causes the authors to advise routinely monitoring of preoperative iron status.

In general, guidelines and literature stress the high therapeutic potential of iron therapy in patients with iron deficiency anaemia to increase preoperative haemoglobin level, to lower the need for blood transfusions and to improve short-term postoperative outcomes. An important caveat raised by ESMO is that oral—as opposed to intravenous—iron administration is quite ineffective in, as our study shows, the major part of patients that have some form of FID. Inflammation-related IL-6 increased hepcidin production namely hampers iron absorption from the duodenum [8, 24]. Furthermore, there is an increased uptake and retention of iron in macrophages, resulting in limitation of availability of iron for iron-restricted erythropoiesis.

Notwithstanding its increased efficacy, timing and dosing are crucial for intravenous iron therapy. Maximal Hb response namely usually takes 4 to 6 weeks [25], while often more than one dose, maximum of 1 g weekly (i.e. Ferinject or Monofer), is required. As highlighted in our study, such an approach is well feasible for patients with rectum tumours; however, for patients with colon tumours with only on average a 5-week period between diagnosis and surgery, this would be quite a challenge. Furthermore, preoperatively, anaemia was found in almost half of all colon cancer patients, compared to only 20% of rectum cancer patients. However, surgery-mediated blood loss and decrease in Hb level was substantially higher in rectum cancer patients, with an increase in postoperative anaemia to 66%, compared to 77% in colon cancer patients. This finding suggests that an even more proactive approach to correct preoperative anaemia in all rectal cancer patients seems to be warranted.

An additional comment, however, should be made. Despite the increased use and success of preoperative—often intravenous—iron therapy to correct anaemia, there are no clinical studies addressing long-term effects of iron therapy in colorectal cancer patients. The importance of this is highlighted by the fact that iron is an important growth factor for rapidly proliferating cells including bacteria and tumour cells. FID in this regard is believed to be a potentially effective defense strategy of the human body to inhibit the growth of pathogens. Several experimental animal studies have shown that exposure to iron can be a risk factor for developing colorectal cancer and tumour growth [10, 26, 27]. While oral iron might induce intraluminal tumour growth, intravenous iron could in this respect additionally be a potential risk for stimulating growth of metastases.

Ultimately, in preoperative blood management, the potential risks of blood transfusion and iron supplementation have to be cautiously weighed up against the risks of anaemia and iron deficiency. Importantly, concerning oncological patients, not only short-term but also long-term oncological effects have to be included in this risk assessment. Preoperative anaemia and blood transfusion have already been strongly associated with a worse oncological outcome [5, 28]. The oncological effects of iron supplementation, however, have not been studied yet. Therefore, clinical studies comparing the long-term effects of anaemia and iron deficiency with the long-term effects of iron supplementation and blood transfusion are required to establish the optimal blood management strategy in oncological patients.

### Strengths and limitations

One of the strengths of the present study is the timing of measuring iron status of patients. Iron status was measured directly after colonoscopy, where a lesion suspicious of colorectal cancer was noticed. As a result, in the vast majority, the iron status we used was not yet affected by any iron supplementation and therefore a reliable representation of condition around diagnosis. The major limitation of this study was the sample size. Therefore, in comparing characteristics of AID and FID, and in assessing the association between iron deficiency and postoperative complication, the small sample size did not allow us to draw firm conclusion on associations. In addition, nonetheless, up till now, this is the largest group of colorectal cancer patients in which the prevalence and type of iron deficiency are described.

## Conclusion

This study shows a high prevalence of preoperative iron deficiency in colorectal cancer patients, including a high percentage of patients with—a component of—functional iron deficiency, and frequently associated an increased postoperative complication rate, anaemia, right-sided colon tumours, advanced age and tumour stage, and poor physical status. As both types of iron deficiency require a different treatment strategy, our results illustrate the therapeutic potential of especially intravenous iron supplementation in patients with severe iron deficiency and stress the urgency of routinely monitoring preoperative iron status and differentiation between types of iron deficiency. As iron therapy may also be potentially harmful in respect to stimulation of tumour growth, future clinical trials assessing the long-term effect of iron therapy are necessary.
